# Associations between lyric and musical depth in Chinese songs: Evidence from computational modeling

**DOI:** 10.1002/pchj.785

**Published:** 2024-06-19

**Authors:** Liang Xu, Bingfei Xu, Zaoyi Sun, Hongting Li

**Affiliations:** ^1^ Department of Psychology, College of Education Zhejiang University of Technology Hangzhou China

**Keywords:** audio, lyric, machine learning, musical depth

## Abstract

Musical depth, which encompasses the intellectual and emotional complexity of music, is a robust dimension that influences music preference. However, there remains a dearth of research exploring the relationship between lyrics and musical depth. This study addressed this gap by analyzing linguistic inquiry and word count‐based lyric features extracted from a comprehensive dataset of 2372 Chinese songs. Correlation analysis and machine learning techniques revealed compelling connections between musical depth and various lyric features, such as the usage frequency of emotion words, time words, and insight words. To further investigate these relationships, prediction models for musical depth were constructed using a combination of audio and lyric features as inputs. The results demonstrated that the random forest regressions (RFR) that integrated both audio and lyric features yielded superior prediction performance compared to those relying solely on lyric inputs. Notably, when assessing the feature importance to interpret the RFR models, it became evident that audio features played a decisive role in predicting musical depth. This finding highlights the paramount significance of melody over lyrics in effectively conveying the intricacies of musical depth.

## INTRODUCTION

Music plays a significant role in human life as it enables the expression of emotions, preservation of culture, and facilitates social interaction (Mehr et al., 2019). In the digital music age, the emergence of algorithmically governed streaming services has greatly influenced people's daily music listening behavior (Fricke et al., 2021). Music information retrieval systems utilize high‐level perceptual dimensions to categorize music effectively (Pesek et al., 2017), and music recommendation systems rely on users' past behavior to suggest music that aligns with their preferences (Kim et al., 2019; Magron & Févotte, 2021). Hence, the psychological structures underlying individual musical preferences have garnered considerable scholarly attentions (Anderson et al., 2021; Bauer & Schedl, 2019; Xu, Zheng, et al., 2021). Over the past decade, a series of studies have confirmed the existence of three stable dimensions of music perception—arousal, valence, and depth—linked to music preference (Fricke et al., 2018, 2021; Fricke & Herzberg, 2017; Greenberg et al., 2016). However, compared to valence and arousal, the concept of musical depth has received relatively less attention.

Musical depth, characterized by the intellectual and emotional complexity of music (Greenberg et al., 2016), has been established as a robust dimension of music perception. Greenberg et al. (2016) were the first to propose that depth is as crucial as valence and arousal in music perception. This three‐dimensional structure has been consistently identified in studies utilizing self‐report assessments (Fricke & Herzberg, 2017) as well as computer‐based music feature analysis (Fricke et al., 2018). Leveraging big data from streaming services, recent research further supports the stability of this three‐dimensional structure (Fricke et al., 2021). In this study, drawing upon prior research on musical depth (Greenberg et al., 2016), we conceptualize musical depth as a psychological attribute of perception, characterized by traits including thoughtfulness, complexity, wisdom, sophistication, inspirational, poetic, and emotionally rich qualities. Previous studies have primarily focused on examining the robustness of the depth dimension and its association with music preference. Some studies have also explored individual differences in this relationship (Basiński et al., 2021; Maroely & Munichor, 2022). However, there remains a paucity of research investigating the connection between musical structure and musical depth.

Establishing the mapping relationship between musical structure (characterized by low‐ and mid‐level music features) and the perceptual attributes of music is a crucial direction in music research. In the realm of music emotion, studies have made significant strides in understanding the link between music features and the perceived or felt emotions of music (Juslin & Laukka, 2004; Xu, Wen, et al., 2021). These connections not only enhance our comprehension of music cognition patterns (Gabrielsson, 2016; Temperley, 2004) but also find practical applications in domains such as music emotion recognition and music information retrieval (Yang et al., 2018). However, when it comes to musical depth, only one recent study has explored the relationship between low‐level audio features and the perceived depth of music (Wen et al., 2022). This study revealed a close association between spectral contrast in audio and musical depth, suggesting that deep music tends to exhibit clear and narrow‐band audio signals. While there have been studies investigating the relationship between depth‐related concepts and musical features, such as Nagathil et al's. (2018) construction of a music feature‐based linear regression model to predict the level of difficulty in following and understanding music, and Rigg's (1964) exploration of music features in dreamy music (dreamy is a sub‐attribute of musical depth; Greenberg et al., 2016), an important element of music—lyrics—has been overlooked.

Melody and lyrics, representing two distinct cognitive abilities, are commonly combined in music to convey thoughts and feelings (Gordon et al., 2010). While the processing of melodic and lyrical components occurs independently in the brain (Besson et al., 1998), lyrics are often seen as a supportive element that aids the melody in transmitting information (Ali & Peynircioğlu, 2006; Ma et al., 2023; Serafine et al., 1986). The investigation of the interaction or independence between melody and lyrics has remained a continuous and vital focus of research (Kolinsky et al., 2009). Behavioral studies have contributed to clarifying the role of lyrics in conveying ideas, emotions, and other information by examining the effects of lyrics in various contexts (Ali & Peynircioğlu, 2006; Gordon et al., 2010; Yu et al., 2019). Computational studies have also explored the impact of lyrics by comparing the performance of music recognition models with and without lyric features (Hu et al., 2009; Laurier et al., 2008; Xu, Sun, et al., 2021). Moreover, evidence from electroencephalogram (EEG) and functional magnetic resonance imaging (fMRI) studies has underscored the significance of lyrics in information processing during musical experiences (Brattico et al., 2011; Proverbio et al., 2020). Hence, when it comes to musical depth, do the roles of melody and lyrics align with the findings of previous studies? What is the relationship between the perception of musical depth and the information conveyed through lyrics? This study aims to explore the above questions.

Another vital question concerns the operationalization of lyric information. Traditionally, lyrics have been treated as a holistic entity in behavioral research. Studies often focused on examining the differences between music with lyrics and instrumental music (Ali & Peynircioğlu, 2006; Yu et al., 2019) or investigated the effects of lyrics conveying specific meanings, such as nostalgia (Batcho et al., 2008), happiness, and sadness (Brattico et al., 2011). However, there has been limited analysis of the elements extracted from lyrics. In other words, the emphasis of previous behavioral research was on the overall presence or absence of lyrics and the overall semantic content they conveyed. The nuanced elements within the lyrics themselves were not extensively explored or analyzed. Recent advancements in natural language processing (NLP) technology have allowed for the usage of various explainable or inexplicable lyric features in psychological studies (Greenberg et al., 2021; Mori, 2022). NLP techniques, such as text summarization (Fell et al., 2023), word frequency analysis (Xu, Sun, et al., 2021), and word clustering (Vásquez‐Leon & Ugarte, 2020), have greatly contributed to the analysis of lyric content. In light of this, our study utilizes NLP approaches to quantitatively investigate lyric content by extracting relevant features from lyrics.

In the present work, we conducted an exploratory study to investigate the relationship between musical depth and lyric features. This study has two main research objectives. First, we aim to investigate the direct associations between different lyric features and the perceived depth of music. To achieve this, we utilize NLP approaches to extract interpretable lyric features and examine their association with musical depth. Second, we aim to determine the power of audio and lyric features in predicting musical depth and identify the most important features. To accomplish this, we employ machine learning techniques to explore the audio and lyric features associated with musical depth. Furthermore, we perform model explanations by calculating the feature importance, which enables us to compare the relative power and influence of different features in predicting musical depth.

## METHOD

### Data

This study utilized the PSIC3839 dataset (Xu et al., 2022), which is a publicly available dataset comprising 3839 popular songs in China. The dataset includes manually annotated arousal, valence, and depth values, as well as the original music files.

To complement the data, we downloaded the lyrics of songs from NetEase Cloud Music (https://music.163.com/), a popular music site in China. We found that among these 3839 songs, there were songs in languages other than Chinese, such as English, Japanese, and Korean. Considering that the annotators of this database are Chinese, after supplementing the lyrics information for this database, we removed songs in languages other than Chinese and retained only the 2372 songs with Chinese lyrics for analysis.

In addition, this study is a retrospective analysis of publicly available data. The Research Ethics Committee has confirmed that no ethical approval is required. All the data collection and analysis methods were carried out in accordance with relevant guidelines and regulations. There were no human participants in this study, and therefore no corresponding informed consent was required.

### Measurement of musical depth

The dependent variable in this study is the musical depth, which also serves as the true value for the machine learning (ML) models. In the PSIC3839 dataset (Xu et al., 2022), the musical depth values were annotated by 87 Chinese college students, with 61.2% of them being female with an average age of 21.92 ± 2.03 years. For the assessment of depth, participants were instructed to evaluate the entire song. Prior to the annotation process, participants were provided with the meaning of the word “depth” and were given descriptions of the corresponding perceptual attributes based on previous findings (Greenberg et al., 2016). They were instructed that high‐depth music can be characterized as intelligent, sophisticated, inspiring, complex, poetic, dreamy, thoughtful, and emotional, while low‐depth music can be characterized as party music and danceable. The musical depth was measured using a 5‐point Likert scale ranging from −2 (*not at all*) to 2 (*very much*). For example, music perceived as lacking any depth and unrelated to perceptions such as intelligent, sophisticated, inspiring, complex, poetic, dreamy, thoughtful, and emotional was labeled as −2.

Given the large number of songs in the dataset, each participant listened to a subset of the 2372 songs, with each song being evaluated five to seven times. During the formal experiment, participants listened to the music in a predetermined (pseudo‐random) order. Following each song, participants were required to assess the perceived depth of the entire piece. To minimize external distractions and enhance their focus on the music, participants engaged in listening tasks with their eyes closed in a quiet environment. The average annotation result was then considered to be the perceived depth of each song. Additionally, the musical depth values were scaled to a range of 0–1 using min‐max scaling (Kahng et al., 2002) for subsequent analysis.

### Lyric feature extraction

The lyric features serve as the primary independent variables in this study. They are the focus of investigation and analysis, as they carry essential information about the textual content of the songs being examined. To extract the lyric features, the lyrics data first underwent a cleaning process. Initially, the raw lyrics data obtained from online sources contained extraneous information such as singer, composer, and lyricist names. These unwanted elements were manually filtered out. Additionally, since Chinese text does not have explicit word boundaries, word segmentation was performed. This study utilized the Chinese word segmentation tool available at the Language Technology Platform (Che et al., 2010) to segment the text. By implementing these steps, the raw lyrics of each song were processed into a sequence of Chinese words in the correct order for further analysis.

After preprocessing the text data, we employed NLP techniques to analyze the lyric texts. To extract interpretable lyric features, we utilized the word frequency analysis method based on the linguistic inquiry and word count (LIWC) dictionary (Petrie et al., 2008). Referring to previous studies (Alaei et al., 2022; Cui et al., 2021), this approach calculates the usage percentage of different word types, such as causal words, emotional words, and cognitive words, from the LIWC dictionary. The LIWC framework assumes that words convey mental information beyond their literal meaning and can be quantitatively analyzed independent of semantics and context (Petrie et al., 2008). In this study, we utilized the simplified Chinese version of LIWC (SC‐LIWC; Gao et al., 2013; Zhao et al., 2016) to extract lyric features. A total of 98 types of lyric features were retained for each song, including features like *Sad*, which reflects the proportion of words related to sadness (e.g., *agony*, *cry*, and *heartbreak*) in each song. The complete list and descriptions of all extracted features can be found in Table S1.

### Audio feature extraction

In this study, audio features were considered to be important inputs for the ML models. Considering the close relationship between musical depth and emotion, the acoustic features in this study were primarily informed by previous work in music emotion (Gabrielsson & Juslin, 2003) and music signal processing (McFee et al., 2019; Yang et al., 2018). Maintaining consistency with prior research (Wen et al., 2022), a total of nine types of audio features were selected and categorized into three domains (Panda et al., 2020): tonal color, harmony, and rhythm (Table 1).

**TABLE 1:** Audio features used in this study.

Musical domain	Audio feature	Descriptions
Tonal color	MFCCs	Mel‐frequency cepstral cofficients, the measures of spectral shape.
	Spectral centroid	Brightness of the sound (Panda et al., 2020).
	Spectral bandwidth	Compute p'th‐order spectral bandwidth (FitzGerald & Paulus, 2007).
	Spectral roll‐off	Metrics for the amount of high‐frequency energy in the signal.
	Spectral flatness	Smooth (or spikyness) of data.
	Spectral contrast	The spectral contrast of a spectrum.
Harmony	Tonal centroid features	Harshness among tonal components (Harte et al., 2006).
	Chromagram	Energy distribution along pitches.
Rhythm	Tempo	Estimated tempo of the music piece.

To extract the audio features, we first conducted audio signal preprocessing. The songs were sampled at a rate of 22,050 Hz, and a short‐term Fourier transform was applied to obtain the power spectrogram. Referring to prior research (Wen et al., 2022), we utilized the librosa package (McFee et al., 2015) to extract eight high‐dimensional features, including Mel‐frequency cepstral cofficients (MFCCs), spectral centroid, spectral bandwidth, spectral roll‐off, spectral flatness, spectral contrast, tonal centroid features, and chromagram. We also estimated the tempo of each song. To reduce the dimensionality of the high‐dimensional features, we performed principal component analysis (PCA) for each type of feature. After PCA, we selected the top 50 dimensions of each audio feature and combined them as inputs for the models. Additionally, we scaled each continuous audio feature to a value between 0 and 1 using min‐max scaling. By employing these procedures, we obtained the relevant audio features that will be used as inputs in our models.

### Analysis strategy

The primary aim of this study is to investigate the direct connections between various lyric features and musical depth. To accomplish this objective, we employed a two‐pronged analytical approach. First, we conducted correlation analyses to explore the relationships between lyric features and musical depth, thereby comparing the effect sizes to identify the features that are closely linked to musical depth. Second, we utilized random forest regression (RFR), a widely employed machine learning technique for similar purposes (Xu, Wen, et al., 2021), to assess the significance of lyric features. By combining predictions from multiple decision trees, RFR enables the identification of nonlinear relationships between the input variables and the ground truth. The derived feature importance from these decision trees provides valuable insights into the associations between lyrical features and musical depth. Reporting the results of both statistical analysis and machine learning analysis can help us gain a more comprehensive understanding of the complex relationships between variables. This approach has been widely employed in psychology research in recent years (e.g., Xu, Zeng, et al., 2023).

In the above step, we utilized all the lyric features as inputs and the musical depth values as the ground truth to construct regressions using the RFR algorithm. To enhance the performance of our models, we conducted a comprehensive grid parameter search to identify the optimal parameter settings. The Gini importance metric (Strobl et al., 2009) was employed to evaluate the explanatory power of the input variables in each regression. To mitigate the potential for overfitting, we employed tenfold cross‐validation. This approach guards against biased results by dividing the dataset into 10 subsets. During each iteration, nine subsets were utilized for training the models, while the remaining subset was reserved for testing. Subsequently, the feature importance was calculated 10 times for predicting musical depth. It is worth noting that the absolute scores of feature importance do not possess inherent meaning; rather, the primary objective of this step is to establish a relative ranking and comparison among the predictor variables.

**FIGURE 1:**
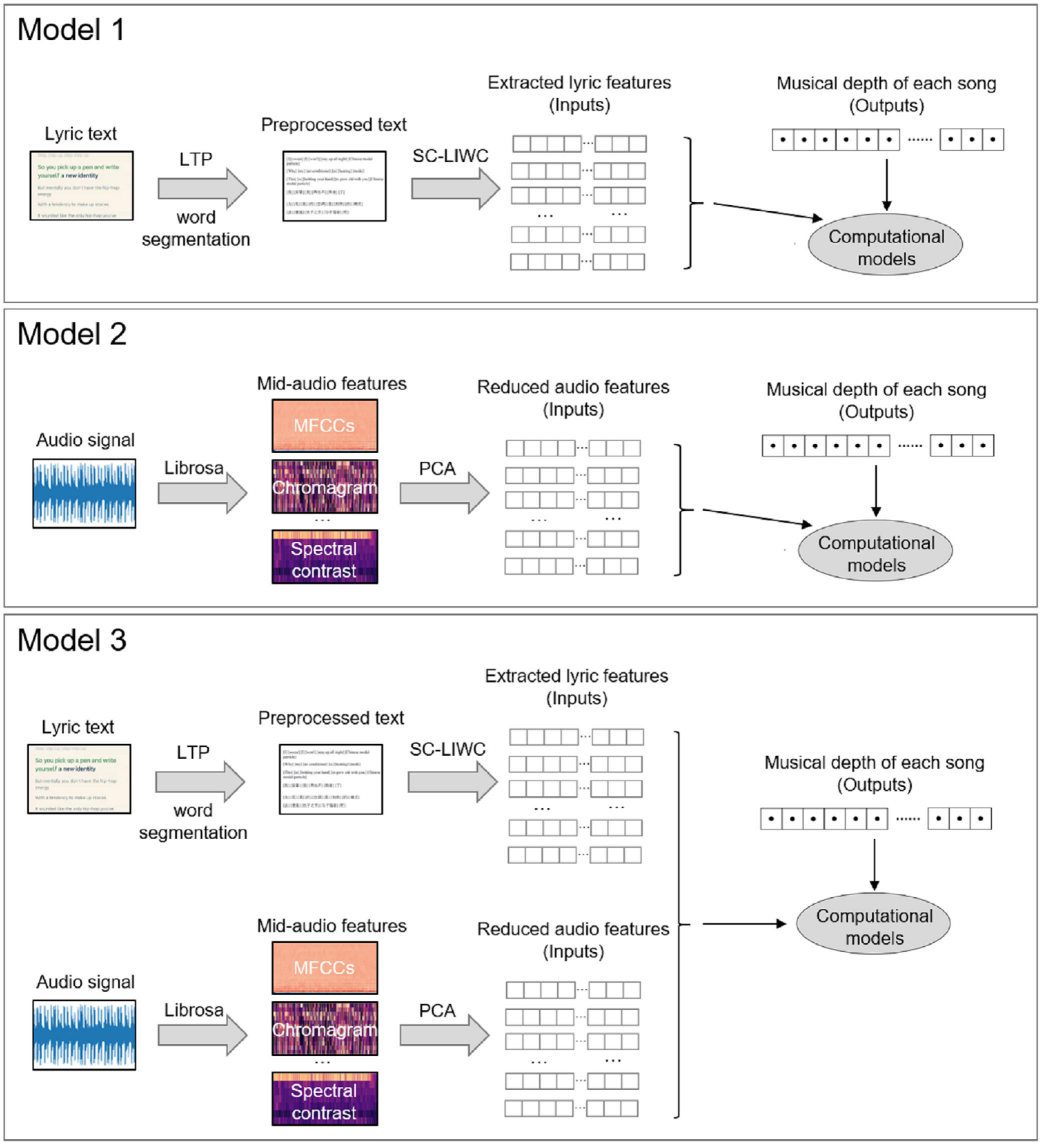
The model construction process.

The second objective of this study is to evaluate and compare the significance of audio and lyric features in predicting musical depth. To achieve this, we employed the RFR algorithm to construct three distinct types of music depth prediction models. The first model utilized only lyric features as input, the second model used only audio features, and the third model incorporated both types of features (see Figure 1). By analyzing and comparing the prediction performance of these models, we can gain valuable insights into the specific contributions of lyric and audio features in predicting musical depth. To assess the accuracy of the model predictions, we employed the *R* ^2^ statistics and the root‐mean‐square error (RMSE). In the subsequent step, we also conducted an analysis of feature importance in the RFR models to assess and compare the predictive power of all the lyric and audio features.

## RESULTS

### Differences in lyric content

**FIGURE 2:**
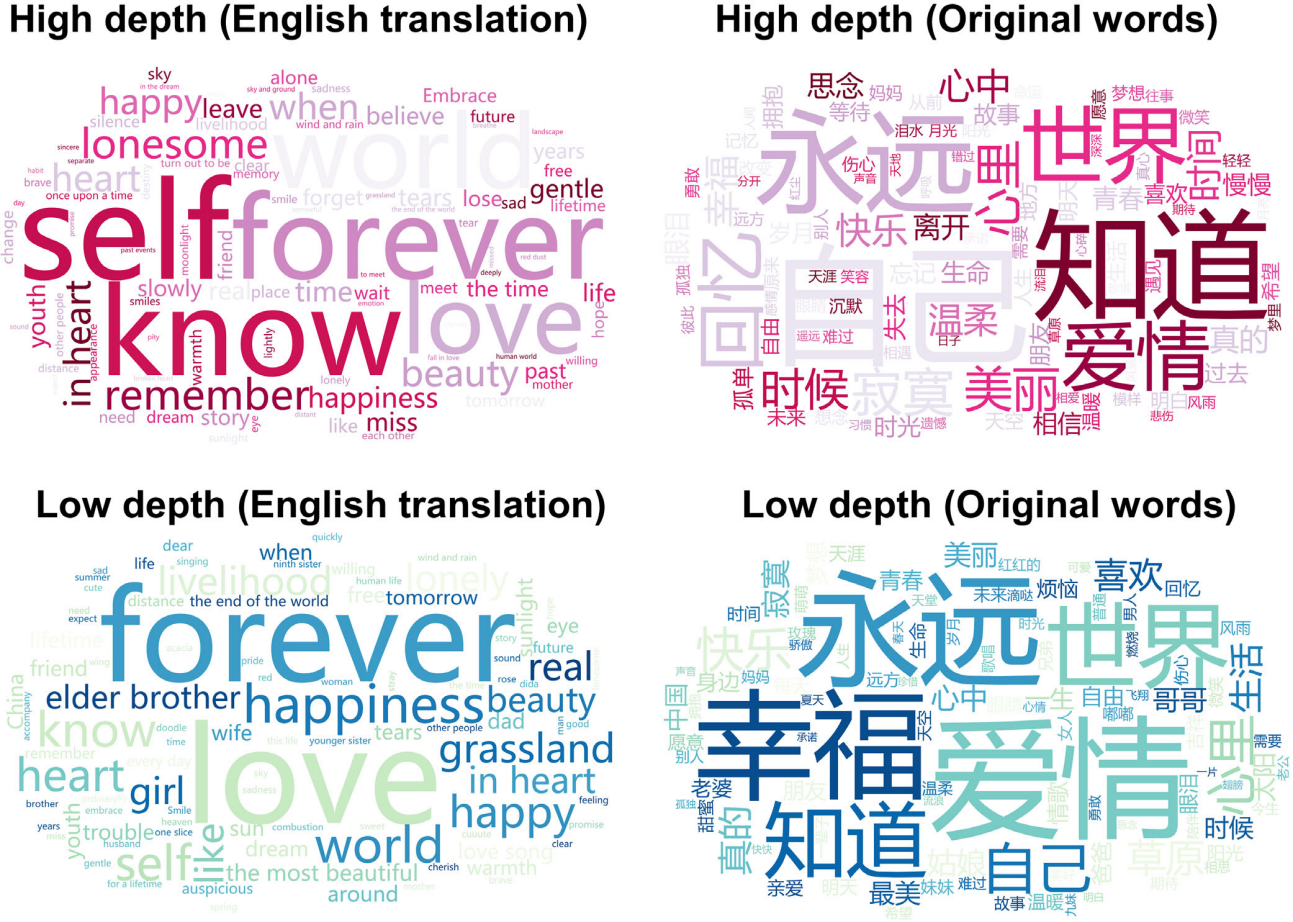
Word cloud of top 100 words in lyrics of high depth and low depth music.

To initiate the data exploration process, we conducted a comparison to determine if there are variations in word usage between high and low depth music. The word cloud (Figure 2) provides a visual representation of these findings. It is noteworthy that certain words are commonly used regardless of the depth of the song, such as *forever*, *world*, *know*, and *love*. However, distinctions between the two categories are also evident. For example, in high‐depth music, words like *lonesome*, *remember*, *lose*, and *leave*, appear more frequently. Conversely, low‐depth music tends to employ family‐related words such as *brother* and *sister* with greater frequency.

**FIGURE 3:**
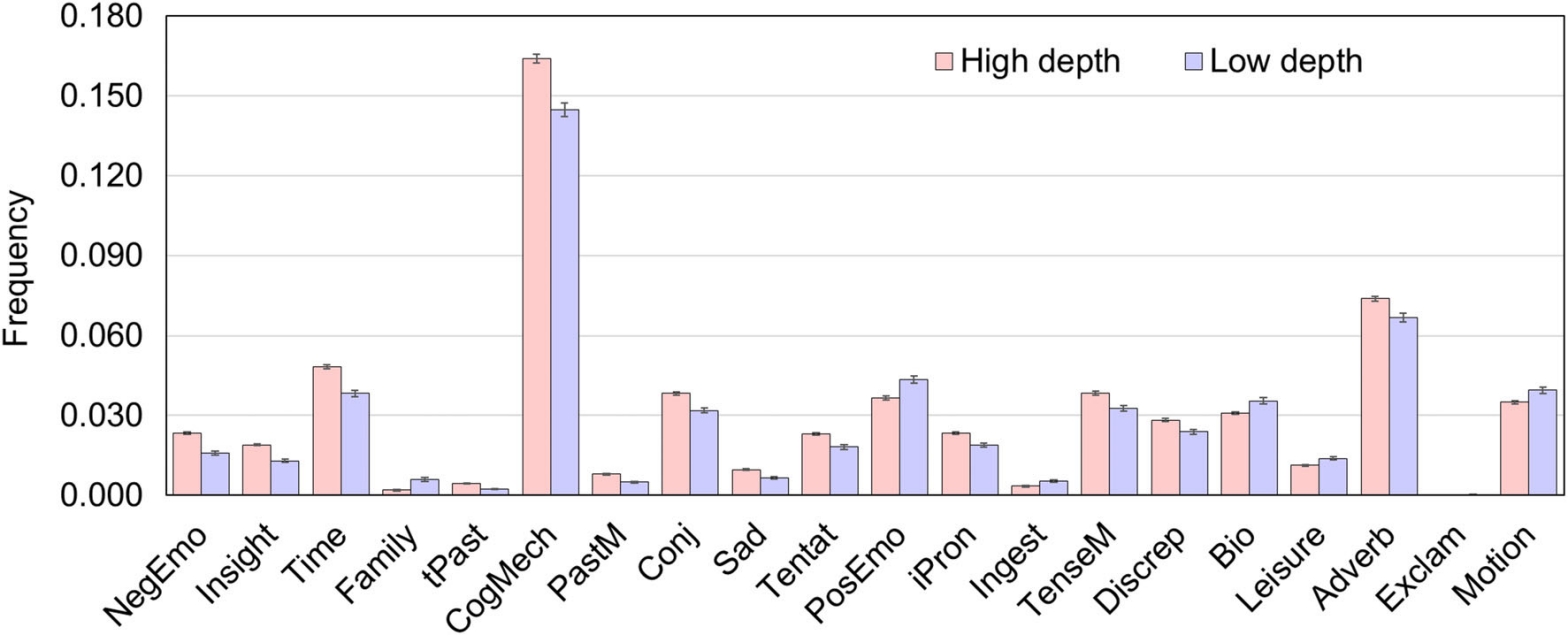
Differences in the lyric features between the high‐depth and low‐depth music. This figure presents the 20 most significant differences in lyric features, while the differences in the remaining lyric features are provided in Table S2.

We classified all songs into two groups, high depth and low depth, based on the scores of perceived depth, using a cutoff score of zero. Then, we used SC‐LIWC to calculate the frequency of different types of words, obtaining the lyric features. Subsequently, we conducted a comparison to examine the disparities between high and low depth music for each lyric feature. The Kolmogorov–Smirnov test indicated that the samples did not follow a normal distribution. Therefore, we employed the Mann–Whitney U‐test to compare the usage frequency of lyrical features between high and low depth music. Figure 3 illustrates the 20 most significantly different lyric features. Our findings indicate that, for example, compared to low‐depth music, high‐depth music incorporates a higher usage of negative emotion words (*Z* = 9.658, *p* < .001, *r* = 0.203), words related to insight (*Z* = 8.975, *p* < .001, *r* = 0.189), and words associated with time (*Z* = 8.416, *p* < .001, *r* = 0.177). Conversely, low‐depth music tends to utilize more words related to family (*Z* = 8.443, *p* < .001, *r* = 0.178), positive emotion words (*Z* = 4.330, *p* < .001, *r* = 0.091), and words associated with ingest (including food and actions associated with eating, such as *sandwich*, *tea*, *meal*, *eat*, and *swallow*) (*Z* = 4.040, *p* < .001, *r* = 0.085) than high‐depth music. These results further affirm that there are indeed distinctions in the choice of words within the lyrics of high‐ and low‐depth music. In addition, the comparison of differences in all lyric features between high‐depth and low‐depth music can be found in Table S2.

### Correlation analysis

To investigate the direct connections between lyric features and musical depth, we initially conducted correlation analyses between all lyric features and musical depth. Correlation analysis results also serve as a crucial supplement to subsequent ML feature importance analysis, aiding in determining the positive or negative direction of the effects of predictors on the predicted outcome. Table 2 presents the lyric features that exhibit the highest relevance to musical depth. For instance, based on Pearson correlation analysis, the depth values were found to have a positive correlation with the proportion of words related to negative emotions (*r*[2371] = 0.155, *p* < .01), the proportion of words related to the past (*r*[2371] = 0.155, *p* < .01), and the proportion of words related to time (*r*[2371] = 0.150, *p* < .01). On the other hand, the depth values showed a negative correlation with the proportion of words related to family (*r*[2371] = −0.163, *p* < .01), the proportion of words related to positive emotions (*r*[2371] = −0.145, *p* < .01), and the ratio of Latin words (*r*[2371] = −0.130, *p* < .01). Complete correlation results can be found in Table S3. Collectively, these findings indicate the existence of a linear relationship between lyric features and musical depth. To reinforce the findings, we further employed ML methods as a complementary analysis.

**TABLE 2:** Correlation between lyric features and musical depth.

	1	2	3	4	5	6	7	8	9
Musical Depth	1.000								
Family	−.163***	1.000							
NegEmo	.155***	−.102***	1.000						
tPast	.155***	−.057**	0.038	1.000					
Time	.150***	−.091***	−0.009	.322***	1.000				
Insight	.147***	−.097***	.173***	.153***	.139***	1.000			
PastM	.145***	−.083**	.098***	.517***	.299***	.168***	1.000		
PosEmo	−.145***	.032**	.042*	−.055**	−0.039	.091***	−.045*	1.000	
RateLatinWord	−.130***	−.032	−0.009	−0.031	−0.032	0.020	−0.035	0.016	1.000

*Note*: This table presents only the top 10 features with the highest correlations, while the complete results of the correlation analysis can be found in Table S3.

* *p* < .05;

** *p* < .01;

*** *p* < .001.

### Results from RFR models with lyric features

In this phase, we utilized lyric features as inputs to construct prediction models for musical depth, and the feature importance derived from the constructed RFR models provided insights into the nonlinear relationship between lyric features and musical depth. Overall, the constructed prediction models yielded a mean *R* ^2^ value of 0.225 ± 0.050 and a mean RMSE value of 0.210 ± 0.012, indicating that lyric features have a moderate predictive capacity for musical depth.

**FIGURE 4:**
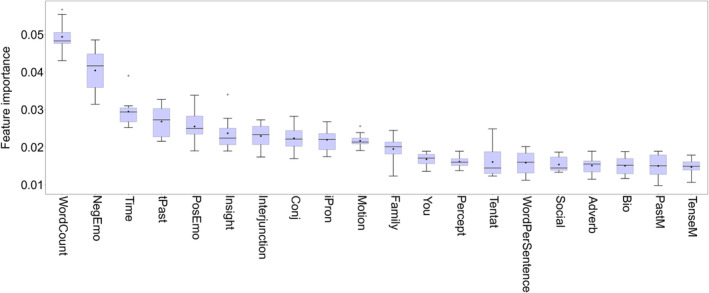
Feature importance distribution of lyric features from the random forest regression models. Boxplots are sorted by the mean values, and only the top 20 features are included for visibility, with trends of the remaining features being approximately the same. The black line in the middle of each box indicates the median value, and the black dot indicates the mean value.

Subsequent analysis of feature importance revealed that the most influential lyric feature, accounting for 4.94% of the models, was the number of words (*WordCount*). Following *WordCount*, other significant features included *NegEmo* (4.05%), *Time* (2.95%), *tPast* (2.68%), and *PosEmo* (2.55%), as depicted in Figure 4. These findings align with the results obtained from the Pearson correlation analyses (see Table S3). Consequently, it is evident that lyric features are indeed associated with the musical depth. Specifically, the number of words and the frequency of specific types of words, such as those pertaining to negative and positive emotions, play a role in influencing the perception of musical depth.

### Results from RFR models with different inputs

To compare the effects of melody‐derived audio features and lyric features on musical depth, we constructed RFR models using audio features as input, lyric features as input, and all features as input, respectively. By comparing the effects of these models, we analyzed the role of audio and lyric features. To identify the optimal parameter settings, we conducted a grid parameter search, and the best performing parameters for each RFR model are presented in Table S3.

**FIGURE 5:**
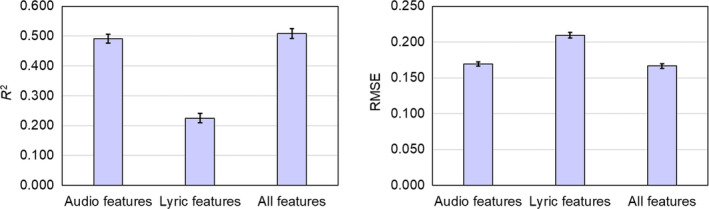
The performance of random forest regression models with different inputs.

The analysis of the RFR models' performance, as shown in Figure 5, indicates a significant difference between the model with only lyric features as input and the model with audio features as input (*R* ^2^: *t*(10) = −13.178, *p* < .001, *d* = −4.393; RMSE: *t*(10) = 9.091, *p* < .001, *d* = 3.030). The model utilizing audio features outperformed the one relying solely on lyric features. Upon incorporating lyric features into the audio‐based model (*R* ^2^ = 0.491 ± 0.047, RMSE = 0.169 ± 0.009), there was a slight improvement in performance (*R* ^2^ = 0.508 ± 0.050, RMSE = 0.166 ± 0.010). However, this improvement was not statistically significant (*R* ^2^: *t*(10) = −0.779, *p* = .456, *d* = 0.260; RMSE: *t*(10) = 0.631, *p* = .544, *d* = 0.210). These findings suggest that melodic information holds greater importance in the perception of musical depth compared to lyric features.

**FIGURE 6:**
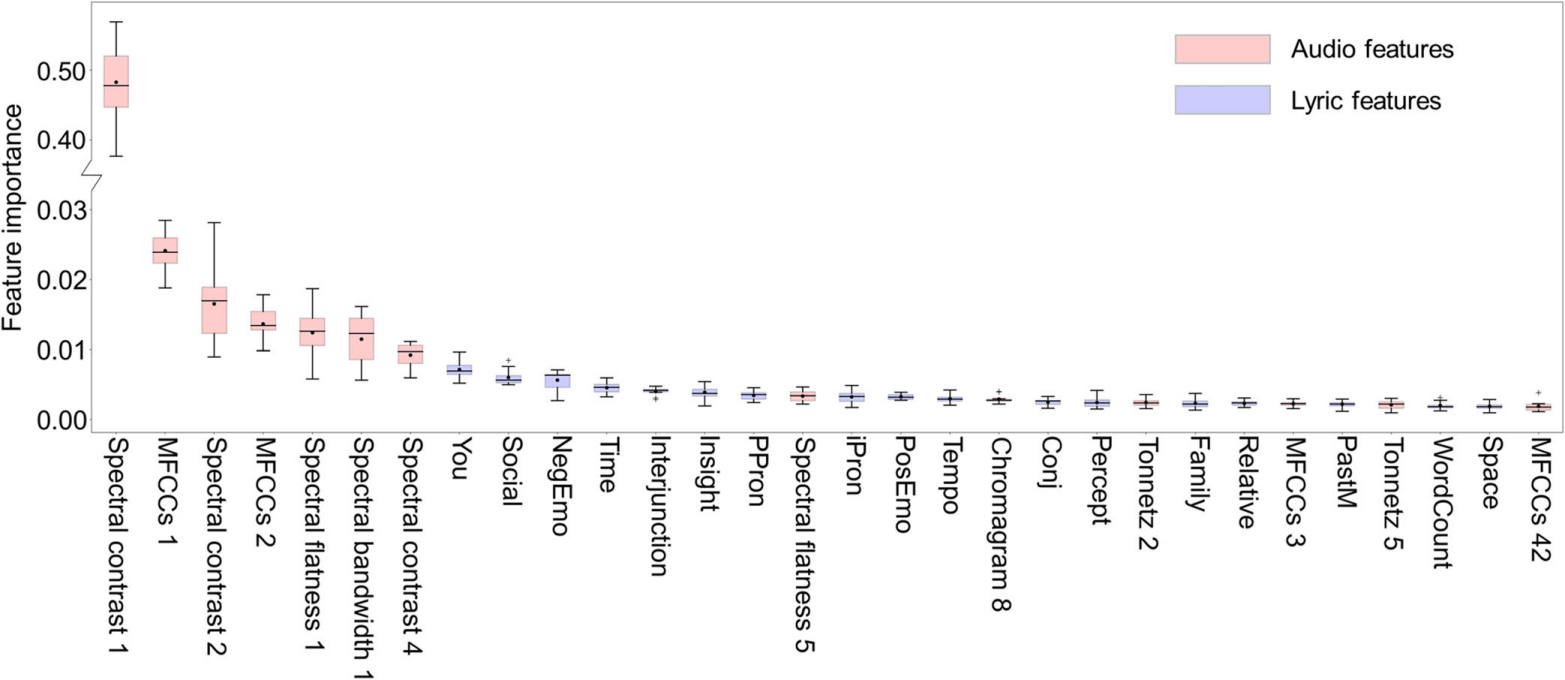
Distribution of feature importance for the random forest regression models with both audio and lyric features. Boxplots are sorted by the mean values, and only the top 30 features are included for visibility, with trends of the remaining features being approximately the same. The black line in the middle of each box indicates the median value, and the black dot indicates the mean value.

We further enhanced our model comparisons by conducting a feature importance analysis for the RFR model using all features. In Figure 6, it is evident that audio features hold a dominant position, accounting for 89.198% of the model. Consistent with previous findings (Wen et al., 2022), the first principal component of spectral contrast emerges as the most crucial feature, explaining 47.148% of the model. The spectral contrast value of the audio reflects whether the music has clear, narrow‐band signals (Jiang et al., 2002). This suggests that in this study, songs perceived as having depth often lack clear, narrow‐band signals. In addition, we found that tonal centroid features, which have been previously highly correlated with music emotion, did not yield satisfactory predictive results. This may be primarily due to the multifaceted nature of musical depth, of which emotional is just one dimension. For instance, songs characterized as “dreamy” are also considered to have depth, and their emotional valence could be either positive or negative. More detailed explanations about audio features can be found in the supplementary materials; Data S1.

Regarding the lyric features, the proportion of second‐person pronouns (*You*) emerges as the most important feature, explaining 0.718% of the model. This is followed by *Social* (0.605%), *NegEmo* (0.565%), *Time* (0.456%), and *Interjunction* (0.405%). The importance distribution of the lyric features slightly differs from the feature importance results of the RFR model using only lyric features. This suggests that the aforementioned lyric features carry greater significance when conveying information in conjunction with the melody. These findings not only reveal greater importance of melody in conveying musical information compared to lyrics (Ali & Peynircioğlu, 2006; Gordon et al., 2010; Ma et al., 2023; Serafine et al., 1986; Yu et al., 2019), but also support the findings of Serafine et al. (1984), indicating that the influence of words such as “you” and “social” is enhanced with increased melody information.

## DISCUSSION

This study employed a data‐driven approach to examine the relationships between lyric features and musical depth, as well as comparing the predictive roles of audio features and lyric features. Combining statistical analysis and ML techniques, we discovered that multivariate lyric features derived from LIWC can effectively predict musical depth. However, it is noteworthy that audio features hold a dominant position in predicting musical depth when compared to lyric features. These findings contribute to the understanding of the mapping relationship between various musical features and musical depth, providing valuable insights for future research.

Our findings reveal both similarities and differences in the lyrics of music with different depths. In terms of similarities, we observed a high usage frequency of words such as *forever*, *world*, *know*, and *love* in both types of music. These words can be considered common vocabulary in songs and are not specifically related to the perception of musical depth. Previous studies suggest that music can reflect the preferences and values of a cultural group (DeWall et al., 2011; Mehr et al., 2019; Savage, 2019; Xu, Xu, et al., 2023). Analyzing the musical characteristics of popular songs can provide valuable insights into the sociocultural traits of a particular context (Jo & Kim, 2022). Additionally, Freeman (2012) has found that love‐themed songs dominate the Chinese pop music scene, aligning with our findings. The observed similarities highlight the influence of sociocultural factors and prevailing themes on the lyrical content of songs, underscoring the importance of considering cultural context when interpreting and analyzing music.

In terms of differences, we observed that deep music exhibited a higher occurrence of negative emotion‐related words such as *loneliness*, *lose*, and *leave*, while low‐depth music featured more positive words. These findings align with previous research indicating that music expressing negative emotions is often perceived as having more depth (Wen et al., 2022). The concept of musical depth encompasses both intellectual and emotional aspects (Greenberg et al., 2016). To understand why negative music is often associated with depth, we can consider two reasons. First, intellectual depth in music refers to its complexity, intricacy, and the depth of musical elements employed within a composition. Negative music tends to involve complex musical structures. For instance, dissonant harmonies are commonly associated with expressions of tension, anger, sadness, and unpleasantness (Gabrielsson, 2016). Similarly, irregular or complex rhythms can convey feelings of uneasiness and anger (Juslin & Laukka, 2004). These intricate musical elements contribute to the perceived intellectual depth of negative music. On the other hand, negative music often has a stronger emotional impact on listeners compared to positive music. Neuroimaging studies using fMRI have shown that sad music, in comparison to happy music, elicits stronger emotional experiences (Brattico et al., 2011), potentially capturing the emotional depth associated with negative music. Of course, the above speculation requires further validation through future research. Exploring the relationship between musical elements, emotional experiences, and perceived depth would provide a deeper understanding of why negative music is often perceived as having more depth.

The results of the correlation analysis and ML modeling provide further evidence of the close relationship between lyric features and musical depth. In addition to the emotional words mentioned earlier, features such as the frequency of insight words (e.g., *know*, *remember*, *notice*) and the frequency of time words (e.g., *tomorrow*, *autumn*, *evening*) are positively correlated with musical depth. These words are often used to describe specific scenarios. For example, the insight words are frequently combined with negative words to create a melancholic atmosphere (Xu, Sun, et al., 2021). Another interesting finding is the higher frequency of family words in low‐depth songs. This observation reflects a phenomenon commonly observed in Chinese pop songs, where certain family words, particularly *brother* and *sister*, are extensively used in a genre known as “unfashionable love songs” (*Tu Wei Qing Ge* in Chinese). These songs are often considered lacking in depth. In summary, it is evident that the choice of words in lyrics is related to the musical depth, as words have the ability to shape perception (Gendron et al., 2012).

Furthermore, our findings highlight that melody‐derived audio features exhibit stronger correlations with musical depth compared to lyric features. This is evident from both the comparison of different ML predictions and the feature importance analysis of the RFR models, both of which consistently support the dominance of audio features in predicting musical depth. This finding aligns with the earlier point that melody information plays a primary role in conveying musical information compared to lyrics (Ali & Peynircioğlu, 2006; Gordon et al., 2010; Ma et al., 2023; Serafine et al., 1986; Yu et al., 2019). From a practical standpoint, to achieve accurate predictions of the perceived depth of music in the future, it will be essential to extract and incorporate more features from the melody when constructing prediction models. By utilizing multivariate audio features in the computational model, we can further enhance our understanding and decoding of the musical depth (Mori, 2022; Wen et al., 2022). This insight opens up avenues for improved depth perception analysis and can contribute to advancements in the field of music research.

This study is subject to several limitations. First, although we examined the influence of LIWC‐based lyric features on musical depth, we did not consider the specific meanings conveyed by the lyrics. The lyric features extracted in this study are individual features that may not capture the full meaning of the entire sentence or paragraph. This limitation also hinders our ability to investigate the underlying mechanisms, such as visual imagery and recall triggered by textual cues in the lyrics (Juslin, 2013). Future research should aim to explore the mechanisms through which lyrics impact perceived depth (Eerola & Vuoskoski, 2013). Second, the music stimuli in this study consisted of complete songs (Xu et al., 2022), and the length of these songs may not be ideal for perceptual research. The perception of musical depth can vary within a song, and splitting the music into segments could help capture these fluctuations. However, it is crucial to find a balance in segment length. If the segments are too short, participants may not have sufficient context to evaluate the musical depth accurately. Similar to the approach taken by Xiao et al. (2008) in studying music emotion, finding the appropriate segment length that strikes a balance between capturing fluctuations in depth and providing sufficient context for evaluation is an important direction for future work. Third, this study did not control for certain latent variables that may influence the perception of musical depth. For instance, previous research has demonstrated that music preference and familiarity can impact individuals' perception of music, with familiar music often leading to increased pleasure and arousal (Daimi et al., 2020; McLachlan et al., 2013). It raises the question of whether familiarity also enhances the perception of musical depth. Therefore, future research should incorporate controls for variables such as music familiarity to better understand their potential effects on the musical depth. Fourth, this study relied solely on a dataset of Chinese songs annotated by Chinese participants. Although the findings of this study are similar to those of previous research (Ali & Peynircioğlu, 2006; Ma et al., 2023), future research should aim to enhance the reliability and robustness of the findings by employing a more diverse range of musical materials, participant demographics, experimental methodologies, and data analysis approaches. Fifth, musical depth encompasses various dimensions, including intellectual and emotional complexity (Greenberg et al., 2016). However, this study only examines the relationship between lyrics, melody, and overall perception of musical depth. Therefore, future research could delve deeper into exploring the relationship between musical features and different sub‐dimensions of musical depth, thereby gaining a more comprehensive understanding of the relationship between them. Finally, our analysis of lyric content is limited to the extraction of word frequency features, which provide a rather constrained representation of textual information (Petrie et al., 2008). For instance, the same word may convey different emotions in different contexts (Peng et al., 2017). Hence, future textual analysis of lyrics could incorporate additional elements, such as metaphors (Ndraha, 2018), allegories (Schulenberg, 1995), or cultural references (characteristics of Chinese).

## CONCLUSION

In conclusion, this study delved into the connections between lyric features and musical depth while comparing the predictive roles of audio features and lyric features in musical depth. By analyzing LIWC‐based lyric features from 2372 Chinese songs, both correlation analysis and ML analysis revealed significant relationships between musical depth and various lyric features, such as the frequency of emotion words, time words, insight words, and more. Subsequently, we constructed prediction models for musical depth using both audio and lyric features as inputs. Our findings indicated that the RFR models incorporating both audio and lyric features exhibited superior prediction performance compared to the ones relying solely on lyric inputs. Furthermore, by assessing the feature importance to interpret the RFR models, we observed that audio features played a decisive role in predicting musical depth. This highlights the paramount significance of melody over lyrics in conveying musical depth.

## CONFLICT OF INTEREST STATEMENT

The authors declare no conflict of interest.

## ETHICS STATEMENT

This is a retrospective analysis of publicly available data. The Zhejiang University of Technology's Research Ethics Committee has confirmed that no ethical approval is required. All the data collection and analysis methods were carried out in accordance with relevant guidelines and regulations. There were no human participants in this study, and therefore no corresponding informed consent was required.

## MATERIALS AND CODE AVAILABILITY

The materials and code can be made available upon reasonable request from the corresponding author.

## Supporting information


**Data S1.** Supporting information.

## Data Availability

The data is accessible at https://github.com/xl2218066/PSIC3839.
