# Supplementary material for: Associations between lyric and musical depth in Chinese songs: Evidence from computational modeling
Source: Psych J. 2024 Jun 19;13(6):915–26. doi: 10.1002/pchj.785 (PMC11608776; doi:10.1002/pchj.785)
Supplement: Supplementary file 1 — Data S1. Supporting information. [file PCHJ-13-915-s001.docx]

**Supplemental Materials**

**Contents**

[**Table S1**. Introduction of SC-LIWC based lyric features in this study. 2](#_Toc166527034)

[**Table S2**. Differences in the lyric features between the high-depth and low-depth music. 5](#_Toc166527035)

[**Table S3**. Correlation results between lyric features and musical depth 8](#_Toc166527036)

[**Table S4**. The best performing params in different random forest regressions. 9](#_Toc166527037)

[**The meaning and distinctions between audio features.** 10](#_Toc166527038)

[**Figure S1**. Interpretation of the first principal components analysis component of spectral contrast 10](#_Toc166527039)

## **Table S1**. Introduction of SC-LIWC based lyric features in this study.

| **Id** | **Feature name** | **Feature description** |
| --- | --- | --- |
| 1 | *Funct* | Proportion of function words, including negative words, quantifier, number, and tense calibration words. |
| 2 | *Pronoun* | Proportion of pronoun. |
| 3 | *PPron* | Proportion of personal pronouns. |
| 4 | *I* | Proportion of first-person singular pronouns. |
| 5 | *We* | Proportion of first-person plural pronouns. |
| 6 | *You* | Proportion of second-person pronouns. |
| 7 | *SheHe* | Proportion of third-person singular pronouns. |
| 8 | *They* | Proportion of third-person plural pronouns. |
| 9 | *iPron* | Proportion of impersonal pronouns. |
| 10 | *Article* | Proportion of articles. |
| 11 | *Verb* | Proportion of verbs. |
| 12 | *AuxVerb* | Proportion of auxiliary verbs. |
| 13 | *enPast* | Proportion of past tense words (English). |
| 14 | *enPresent* | Proportion of present tense words (English). |
| 15 | *enFuture* | Proportion of future tense words (English). |
| 16 | *Adverb* | Proportion of adverbs. |
| 17 | *Preps* | Proportion of preposition. |
| 18 | *Conj* | Proportion of conjunction. |
| 19 | *Negate* | Proportion of negative words. |
| 20 | *Quant* | Proportion of function quantifier. |
| 21 | *Number* | Proportion of number. |
| 22 | *Swear* | Percentage of swear words. |
| 23 | *YouPL* | Proportion of second-person plural pronouns. |
| 24 | *PrepEnd* | Proportion of postposition words. |
| 25 | *SpecArt* | Proportion of specific words. |
| 26 | *QuanUnit* | Proportion of quantity unit words. |
| 27 | *Interjunction* | Proportion of interjunction. |
| 28 | *MultiFun* | Proportion of multi-function words |
| 29 | *TenseM* | Proportion of tense markers. |
| 30 | *PastM* | Proportion of past tense markers. |
| 31 | *PresentM* | Proportion of present tense markers. |
| 32 | *FutureM* | Proportion of future tense markers. |
| 33 | *ProgM* | Proportion of words for continuation. |
| 34 | *Social* | Proportion of words related to social relations, including family, friend, and humans. |
| 35 | *Family* | Proportion of words related to family. |
| 36 | *Friend* | Proportion of words related to friend. |
| 37 | *Humans* | Proportion of words related to humans. |

**Table S1** (continued)

| **Id** | **Feature name** | **Feature description** |
| --- | --- | --- |
| 38 | *Affect* | Proportion of words related to affect, including positive and negative emotion words. |
| 39 | *PosEmo* | Proportion of positive emotion words. |
| 40 | *NegEmo* | Proportion of negative emotion words. |
| 41 | *Anx* | Proportion of words related to anxiety. |
| 42 | *Anger* | Proportion of words related to anger. |
| 43 | *Sad* | Proportion of words related to sadness. |
| 44 | *CogMech* | Proportion of words related to cognition, consisting of words related to insight, cause, discrepancy, tentativeness, certainty, inhibition, inclusiveness, and exclusiveness. |
| 45 | *Insight* | Proportion of words related to insight. |
| 46 | *Cause* | Proportion of words related to cause. |
| 47 | *Discrep* | Proportion of words related to discrepancy. |
| 48 | *Tentat* | Proportion of words related to tentativeness. |
| 49 | *Certain* | Proportion of words related to certainty. |
| 50 | *Inhibition* | Proportion of words related to inhibition. |
| 51 | *Inclusive* | Proportion of inclusive words. |
| 52 | *Exclusive* | Proportion of exclusive words. |
| 53 | *Percept* | Proportion of words related to perception, consisting of words related to vision, hearing, and feeling. |
| 54 | *See* | Proportion of words related to vision. |
| 55 | *Hear* | Proportion of words related to hearing. |
| 56 | *Feel* | Proportion of words related to feeling. |
| 57 | *Bio* | Proportion of words related to biology, consisting of words related to body, health, sexual, and ingest. |
| 58 | *Body* | Proportion of words related to body. |
| 59 | *Health* | Proportion of words related to health. |
| 60 | *Sexual* | Proportion of words related to sexual. |
| 61 | *Ingest* | Proportion of words related to ingest. |
| 62 | *Relative* | Proportion of relative words, consisting of words related to motion, space, and time. |
| 63 | *Motion* | Proportion of words related to motion. |
| 64 | *Space* | Proportion of words related to space. |
| 65 | *Time* | Proportion of words related to time. |
| 66 | *Work* | Proportion of words related to work. |
| 67 | *Achieve* | Proportion of words related to achievement. |
| 68 | *Leisure* | Proportion of words related to leisure. |
| 69 | *Home* | Proportion of words related to home. |
| 70 | *Money* | Proportion of words related to money. |
| 71 | *Religion* | Proportion of words related to religion. |
| 72 | *Death* | Proportion of words related to death. |

**Table S1** (continued)

| **Id** | **Feature name** | **Feature description** |
| --- | --- | --- |
| 73 | *Assent* | Proportion of words related to assent. |
| 74 | *Nonfl* | Proportion of pause superfluous words. |
| 75 | *Filler* | Proportion of superfluous fillers. |
| 76 | *Psychology* | Proportion of words related to psychology. |
| 77 | *Love* | Proportion of words related to love. |
| 78 | *tPast* | Proportion of words related to the past. |
| 79 | *tNow* | Proportion of words related to the present. |
| 80 | *tFuture* | Proportion of words related to the future. |
| 81 | *Period* | Proportion of period. |
| 82 | *Comma* | Proportion of comma. |
| 83 | *Colon* | Proportion of colon. |
| 84 | *SemiC* | Proportion of semicolon. |
| 85 | *QMark* | Proportion of question mark. |
| 86 | *Exclam* | Proportion of exclamation mark. |
| 87 | *Dash* | Proportion of dash. |
| 88 | *Quote* | Proportion of quotation marks. |
| 89 | *Apostrophe* | Proportion of abbreviation. |
| 90 | *Parenth* | Proportion of brackets. |
| 91 | *OtherP* | Proportion of other punctuation. |
| 92 | *WordCount* | The total number of words. |
| 93 | *WordPerSentence* | Average number of words per sentence. |
| 94 | *RateDicCover* | Coverage of SC-LIWC. |
| 95 | *RateNumeral* | Proportion of numbers. |
| 96 | *RateSixLtrWord* | The ratio of Chinese word length greater than or equal to 6. |
| 97 | *RateFourCharWord* | The ratio of Chinese word length greater than or equal to 4. |
| 98 | *RateLatinWord* | The ratio of Latin words. |

## **Table S2**. Differences in the lyric features between the high-depth and low-depth music.

|  | **High depth** | | **Low depth** | | **Z** | **p** | **Effect size r** |
| --- | --- | --- | --- | --- | --- | --- | --- |
|  | **M** | **SD** | **M** | **SD** |  |  |  |
| *Funct* | 0.412 | 0.092 | 0.402 | 0.094 | -2.476 | 0.013 | 0.052 |
| *Pronoun* | 0.103 | 0.05 | 0.105 | 0.056 | -0.355 | 0.723 | 0.007 |
| *PPron* | 0.08 | 0.049 | 0.086 | 0.055 | -2.246 | 0.025 | 0.047 |
| *I* | 0.04 | 0.03 | 0.041 | 0.032 | -0.667 | 0.505 | 0.014 |
| *We* | 0.004 | 0.008 | 0.004 | 0.009 | -0.257 | 0.797 | 0.005 |
| *You* | 0.034 | 0.028 | 0.038 | 0.035 | -1.650 | 0.099 | 0.035 |
| *SheHe* | 0.003 | 0.008 | 0.003 | 0.009 | -0.628 | 0.530 | 0.013 |
| *They* | 0 | 0.002 | 0.001 | 0.005 | -0.720 | 0.472 | 0.015 |
| *iPron* | 0.023 | 0.019 | 0.019 | 0.019 | -6.715 | 0.000 | 0.141 |
| *Article* | 0 | 0 | 0 | 0 | 0.000 | 1.000 | 0.000 |
| *Verb* | 0.125 | 0.046 | 0.119 | 0.048 | -2.878 | 0.004 | 0.061 |
| *AuxVerb* | 0.029 | 0.023 | 0.026 | 0.024 | -3.500 | 0.000 | 0.074 |
| *enPast* | 0 | 0 | 0 | 0 | 0.000 | 1.000 | 0.000 |
| *enPresent* | 0 | 0 | 0 | 0 | 0.000 | 1.000 | 0.000 |
| *enFuture* | 0 | 0 | 0 | 0 | 0.000 | 1.000 | 0.000 |
| *Adverb* | 0.074 | 0.039 | 0.067 | 0.042 | -4.489 | 0.000 | 0.095 |
| *Preps* | 0.051 | 0.025 | 0.048 | 0.026 | -1.840 | 0.066 | 0.039 |
| *Conj* | 0.038 | 0.023 | 0.032 | 0.026 | -6.885 | 0.000 | 0.145 |
| *Negate* | 0.003 | 0.007 | 0.003 | 0.006 | -2.635 | 0.008 | 0.055 |
| *Quant* | 0.017 | 0.017 | 0.018 | 0.019 | -0.050 | 0.960 | 0.001 |
| *Number* | 0.014 | 0.016 | 0.014 | 0.018 | -1.526 | 0.127 | 0.032 |
| *Swear* | 0.001 | 0.002 | 0.001 | 0.002 | -1.102 | 0.270 | 0.023 |
| *YouPL* | 0 | 0.001 | 0 | 0.001 | -1.500 | 0.134 | 0.032 |
| *PrepEnd* | 0.018 | 0.016 | 0.017 | 0.021 | -2.249 | 0.024 | 0.047 |
| *SpecArt* | 0.011 | 0.012 | 0.01 | 0.012 | -1.202 | 0.230 | 0.025 |
| *QuanUnit* | 0.028 | 0.021 | 0.026 | 0.022 | -2.829 | 0.005 | 0.060 |
| *Interjunction* | 0.073 | 0.04 | 0.079 | 0.042 | -2.878 | 0.004 | 0.061 |
| *MultiFun* | 0.073 | 0.038 | 0.075 | 0.041 | -1.062 | 0.288 | 0.022 |
| *TenseM* | 0.038 | 0.026 | 0.033 | 0.026 | -5.684 | 0.000 | 0.120 |
| *PastM* | 0.008 | 0.011 | 0.005 | 0.01 | -8.070 | 0.000 | 0.170 |
| *PresentM* | 0.011 | 0.012 | 0.012 | 0.014 | -0.841 | 0.400 | 0.018 |
| *FutureM* | 0.006 | 0.009 | 0.005 | 0.009 | -5.160 | 0.000 | 0.109 |
| *ProgM* | 0.013 | 0.015 | 0.011 | 0.016 | -3.329 | 0.001 | 0.070 |
| *Social* | 0.08 | 0.045 | 0.087 | 0.054 | -2.734 | 0.006 | 0.058 |
| *Family* | 0.002 | 0.009 | 0.006 | 0.019 | -8.443 | 0.000 | 0.178 |
| *Friend* | 0.002 | 0.007 | 0.003 | 0.009 | -2.471 | 0.013 | 0.052 |
| *Humans* | 0.009 | 0.011 | 0.009 | 0.014 | -1.451 | 0.147 | 0.031 |
| *Affect* | 0.075 | 0.04 | 0.073 | 0.043 | -1.139 | 0.255 | 0.024 |
| *PosEmo* | 0.037 | 0.026 | 0.043 | 0.033 | -4.330 | 0.000 | 0.091 |
| *NegEmo* | 0.023 | 0.021 | 0.016 | 0.019 | -9.658 | 0.000 | 0.203 |
| *Anx* | 0.003 | 0.006 | 0.002 | 0.006 | -3.932 | 0.000 | 0.083 |
| *Anger* | 0.002 | 0.005 | 0.002 | 0.005 | -1.431 | 0.152 | 0.030 |
| *Sad* | 0.01 | 0.012 | 0.006 | 0.011 | -7.796 | 0.000 | 0.164 |
| *CogMech* | 0.164 | 0.064 | 0.145 | 0.066 | -6.662 | 0.000 | 0.140 |
| *Insight* | 0.019 | 0.017 | 0.013 | 0.016 | -8.975 | 0.000 | 0.189 |
| *Cause* | 0.011 | 0.013 | 0.01 | 0.013 | -2.327 | 0.020 | 0.049 |
| *Discrep* | 0.028 | 0.023 | 0.024 | 0.024 | -5.089 | 0.000 | 0.107 |
| *Tentat* | 0.023 | 0.019 | 0.018 | 0.02 | -7.118 | 0.000 | 0.150 |
| *Certain* | 0.012 | 0.012 | 0.011 | 0.013 | -3.071 | 0.002 | 0.065 |
| *Inhibition* | 0.006 | 0.009 | 0.004 | 0.01 | -4.447 | 0.000 | 0.094 |
| *Inclusive* | 0.036 | 0.024 | 0.037 | 0.028 | -0.408 | 0.683 | 0.009 |
| *Exclusive* | 0.031 | 0.023 | 0.027 | 0.026 | -4.544 | 0.000 | 0.096 |
| *Percept* | 0.042 | 0.027 | 0.044 | 0.035 | -0.158 | 0.875 | 0.003 |
| *See* | 0.015 | 0.017 | 0.017 | 0.022 | -0.630 | 0.528 | 0.013 |
| *Hear* | 0.01 | 0.012 | 0.011 | 0.016 | -0.428 | 0.669 | 0.009 |
| *Feel* | 0.009 | 0.011 | 0.008 | 0.014 | -3.145 | 0.002 | 0.066 |
| *Bio* | 0.031 | 0.022 | 0.035 | 0.032 | -1.647 | 0.100 | 0.035 |
| *Body* | 0.012 | 0.012 | 0.013 | 0.02 | -0.138 | 0.890 | 0.003 |
| *Health* | 0.006 | 0.008 | 0.005 | 0.009 | -2.362 | 0.018 | 0.050 |
| *Sexual* | 0.011 | 0.014 | 0.012 | 0.018 | -0.067 | 0.947 | 0.001 |
| *Ingest* | 0.003 | 0.007 | 0.005 | 0.012 | -4.040 | 0.000 | 0.085 |
| *Relative* | 0.135 | 0.051 | 0.128 | 0.055 | -3.641 | 0.000 | 0.077 |
| *Motion* | 0.035 | 0.023 | 0.039 | 0.032 | -1.812 | 0.070 | 0.038 |
| *Space* | 0.064 | 0.035 | 0.065 | 0.041 | -0.502 | 0.616 | 0.011 |
| *Time* | 0.048 | 0.03 | 0.038 | 0.03 | -8.416 | 0.000 | 0.177 |
| *Work* | 0.005 | 0.008 | 0.006 | 0.012 | -1.435 | 0.151 | 0.030 |
| *Achieve* | 0.008 | 0.01 | 0.008 | 0.013 | -1.865 | 0.062 | 0.039 |
| *Leisure* | 0.011 | 0.013 | 0.014 | 0.018 | -1.772 | 0.076 | 0.037 |
| *Home* | 0.002 | 0.006 | 0.002 | 0.005 | -0.450 | 0.652 | 0.009 |
| *Money* | 0.001 | 0.004 | 0.002 | 0.007 | -2.319 | 0.020 | 0.049 |
| *Religion* | 0.007 | 0.012 | 0.006 | 0.011 | -1.579 | 0.114 | 0.033 |
| *Death* | 0.002 | 0.005 | 0.002 | 0.005 | -3.210 | 0.001 | 0.068 |
| *Assent* | 0.031 | 0.023 | 0.033 | 0.028 | -0.638 | 0.524 | 0.013 |
| *Nonfl* | 0.006 | 0.012 | 0.007 | 0.014 | -0.593 | 0.553 | 0.012 |
| *Filler* | 0.006 | 0.009 | 0.007 | 0.013 | -1.664 | 0.096 | 0.035 |
| *Psychology* | 0.025 | 0.018 | 0.022 | 0.019 | -3.692 | 0.000 | 0.078 |
| *Love* | 0.003 | 0.007 | 0.004 | 0.012 | -0.243 | 0.808 | 0.005 |
| *tPast* | 0.004 | 0.007 | 0.002 | 0.006 | -7.591 | 0.000 | 0.160 |
| *tNow* | 0.002 | 0.005 | 0.002 | 0.005 | -2.355 | 0.019 | 0.050 |
| *tFuture* | 0.002 | 0.005 | 0.002 | 0.007 | -1.715 | 0.086 | 0.036 |
| *Period* | 0.001 | 0.006 | 0.001 | 0.005 | -1.669 | 0.095 | 0.035 |
| *Comma* | 0.147 | 0.035 | 0.146 | 0.035 | -0.598 | 0.550 | 0.013 |
| *Colon* | 0 | 0.001 | 0 | 0 | -0.427 | 0.669 | 0.009 |
| *SemiC* | 0 | 0.002 | 0 | 0.002 | -0.360 | 0.719 | 0.008 |
| *QMark* | 0.001 | 0.01 | 0.001 | 0.011 | -0.923 | 0.356 | 0.019 |
| *Exclam* | 0 | 0.001 | 0 | 0.002 | -5.090 | 0.000 | 0.107 |
| *Dash* | 0 | 0.004 | 0 | 0.001 | -2.637 | 0.008 | 0.056 |
| *Quote* | 0 | 0.001 | 0 | 0.001 | -0.448 | 0.654 | 0.009 |
| *Apostrophe* | 0 | 0.002 | 0 | 0.001 | -0.005 | 0.996 | 0.000 |
| *Parenth* | 0.002 | 0.011 | 0.003 | 0.013 | -1.480 | 0.139 | 0.031 |
| *OtherP* | 0.001 | 0.006 | 0.001 | 0.007 | -0.426 | 0.670 | 0.009 |
| *WordCount* | 262.29 | 98.733 | 285.893 | 123.757 | -4.692 | 0.000 | 0.099 |
| *RateDicCover* | 0.84 | 0.096 | 0.828 | 0.091 | -4.136 | 0.000 | 0.087 |
| *RateNumeral* | 0 | 0.002 | 0 | 0.002 | -1.578 | 0.115 | 0.033 |
| *RateSixLtrWord* | 0.001 | 0.012 | 0.002 | 0.006 | -4.253 | 0.000 | 0.090 |
| *RateFourCharWord* | 0.012 | 0.02 | 0.014 | 0.019 | -2.767 | 0.006 | 0.058 |
| *RateLatinWord* | 0.002 | 0.006 | 0.003 | 0.009 | -4.677 | 0.000 | 0.098 |

## **Table S3**. Correlation results between lyric features and musical depth

| **Lyric features r** | | **Lyric features r** | | **Lyric features r** | | **Lyric features r** | |
| --- | --- | --- | --- | --- | --- | --- | --- |
| Funct | 0.034 | Hear | -0.038 | YouPL | -0.035 | Love | -0.041* |
| Pronoun | -0.035 | Feel | 0.008 | PrepEnd | 0.022 | tPast | 0.155** |
| PPron | -0.074** | Bio | -0.093** | SpecArt | 0.039 | tNow | 0.033 |
| I | -0.025 | Body | -0.060** | QuanUnit | 0.040 | tFuture | 0.016 |
| We | -0.053* | Health | 0.031 | Interjunction | -0.078** | Period | 0.008 |
| You | -0.073** | Sexual | -0.049* | MultiFun | -0.041* | Comma | 0.017 |
| SheHe | -0.019 | Ingest | -0.096** | TenseM | 0.125** | Colon | 0.017 |
| They | -0.044* | Relative | 0.076** | PastM | 0.145** | SemiC | -0.006 |
| iPron | 0.104** | Motion | -0.074** | PresentM | 0.003 | QMark | 0.008 |
| Article | - | Space | 0.006 | FutureM | 0.071** | Exclam | -0.068** |
| Verb | 0.019 | Time | 0.150** | ProgM | 0.064** | Dash | 0.054** |
| AuxVerb | 0.057** | Work | -0.058** | Social | -0.098** | Quote | 0.015 |
| enPast | - | Achieve | -0.009 | Family | -0.163** | Apostrophe | 0.002 |
| enPresent | - | Leisure | -0.087** | Friend | -0.059** | Parenth | -0.028 |
| enFuture | - | Home | -0.009 | Humans | 0.007 | OtherP | -0.018 |
| Adverb | 0.063** | Money | -0.071** | Affect | -0.013 | WordCount | -0.115** |
| Preps | 0.057** | Religion | -0.005 | PosEmo | -0.145** | WordPerSentence | -0.080** |
| Conj | 0.120** | Death | 0.035 | NegEmo | 0.155** | RateDicCover | 0.036 |
| Negate | 0.062** | Assent | -0.042* | Anx | 0.049* | RateNumeral | -0.025 |
| Quant | -0.017 | Nonfl | -0.021 | Anger | 0.032 | RateSixLtrWord | -0.051* |
| Number | 0.013 | Filler | -0.090** | Sad | 0.102** | RateFourCharWord | -0.068** |
| Swear | -0.006 | Psychology | 0.055** | CogMech | 0.118** | RateLatinWord | -0.130** |
| Tentat | 0.107** | Percept | -0.044* | Insight | 0.147** | Inhibition | 0.062** |
| Certain | 0.028 | See | -0.043* | Cause | 0.043* | Inclusive | -0.022 |
| Discrep | 0.077** | Exclusive | 0.045* |  |  |  |  |
| **Correlation is significant at the 0.01 level (2-tailed).  *Correlation is significant at the 0.05 level (2-tailed). | | | | | | | |

## **Table S4**. The best performing params in different random forest regressions.

|  | **Model inputs** | | |
| --- | --- | --- | --- |
|  | **Lyric features** | **Audio features** | **All features** |
| n_estimators | 27 | 135 | 179 |
| max_depth | 37 | 20 | 13 |
| min_samples_leaf | 3 | 5 | 3 |
| min_samples_split | 7 | 22 | 15 |
| max_features | 1 | 0.3 | 1 |

## **The meaning and distinctions between audio features.**

In this study, we extracted high-dimensional features from audio data. To reduce the dimensionality of these high-dimensional features, we performed principal component analysis (PCA) for each type of feature. Therefore, all the audio features presented in the final analysis are the results of dimensionality reduction applied to different types of features. In this study, we retained the top 50 PCA components that explained the highest variance for each type of audio feature and sequentially labeled them as feature 1 to feature 50. For instance, *Spectral contrast 1* refers to the PCA component that explains the most variance in the Spectral contrast feature.

For each type of audio feature, the physical interpretation can be elucidated through the reverse decomposition of principal components. For example, Spectral contrast is computed by dividing the spectrogram into seven octave-scale sub-bands (0–200, 200–400, 400–800, 800–1600, 1600–3200, 3200–8000, and >8000 Hz). Therefore, as shown in Figure S1, *Spectral contrast 1* primarily encompasses information related to frequencies in the range of 0–200 Hz or higher than 1600 Hz.


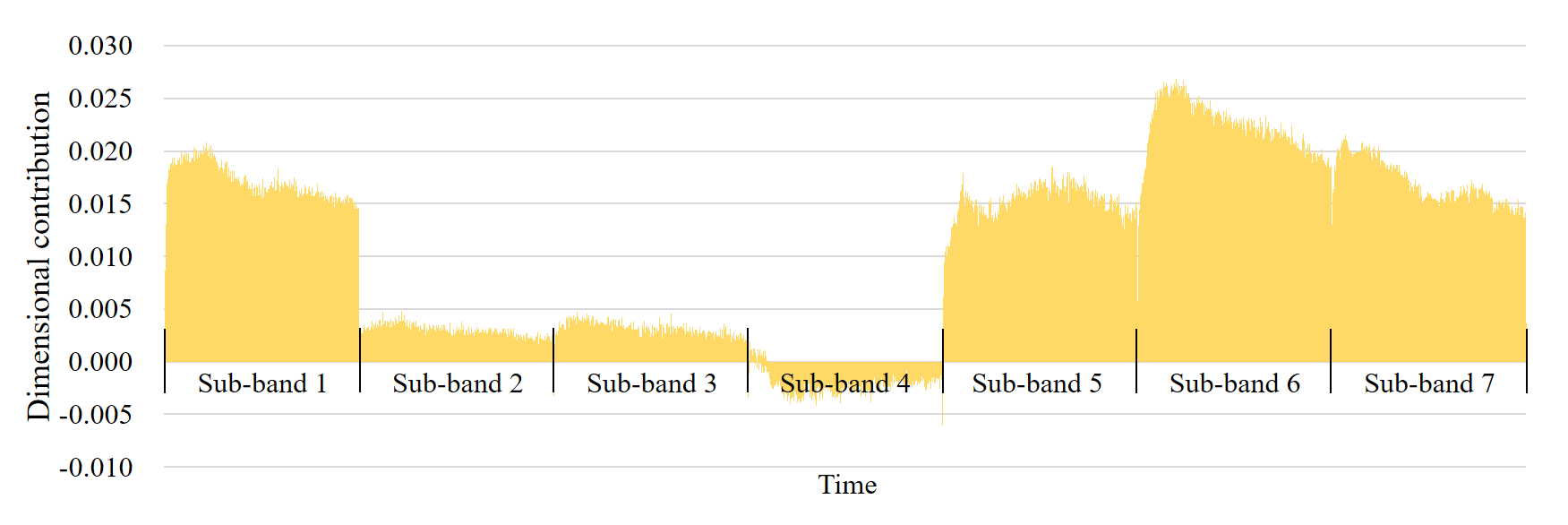


## **Figure S1**. Interpretation of the first principal components analysis component of spectral contrast
